# Collagen VI deficiency causes behavioral abnormalities and cortical dopaminergic dysfunction

**DOI:** 10.1242/dmm.049481

**Published:** 2022-09-21

**Authors:** Ilaria Gregorio, Maddalena Mereu, Gabriella Contarini, Luca Bello, Claudio Semplicini, Francesca Burgio, Loris Russo, Stefania Sut, Stefano Dall'Acqua, Paola Braghetta, Carlo Semenza, Elena Pegoraro, Francesco Papaleo, Paolo Bonaldo, Matilde Cescon

**Affiliations:** ^1^ Department of Molecular Medicine, University of Padova, 35131 Padova, Italy; ^2^ Department of Pharmaceutical and Pharmacological Sciences, University of Padova, 35131 Padova, Italy; ^3^ Genetics of Cognition Laboratory, Neuroscience Area, Istituto Italiano di Tecnologia, 16163 Genova, Italy; ^4^ ERN Neuromuscular Center, Department of Neurosciences, University of Padova, 35129 Padova, Italy; ^5^ IRCCS San Camillo Hospital, 30126 Venice, Italy

**Keywords:** Collagen VI, Mouse model, Central nervous system, Dopamine, Prefrontal cortex, Cognitive function

## Abstract

Mutations of genes coding for collagen VI (COL6) cause muscle diseases, including Ullrich congenital muscular dystrophy and Bethlem myopathy. Although COL6 genetic variants were recently linked to brain pathologies, the impact of COL6 deficiency in brain function is still largely unknown. Here, a thorough behavioral characterization of COL6-null (*Col6a1^–/–^*) mice unexpectedly revealed that COL6 deficiency leads to a significant impairment in sensorimotor gating and memory/attention functions. In keeping with these behavioral abnormalities, *Col6a1^–/–^* mice displayed alterations in dopaminergic signaling, primarily in the prefrontal cortex. *In vitro* co-culture of SH-SY5Y neural cells with primary meningeal fibroblasts from wild-type and *Col6a1^–/–^* mice confirmed a direct link between COL6 ablation and defective dopaminergic activity, through a mechanism involving the inability of meningeal cells to sustain dopaminergic differentiation. Finally, patients affected by COL6-related myopathies were evaluated with an ad hoc neuropsychological protocol, revealing distinctive defects in attentional control abilities. Altogether, these findings point towards a previously undescribed role for COL6 in the proper maintenance of dopamine circuitry function and its related neurobehavioral features in both mice and humans.

This article has an associated First Person interview with the first author of the paper.

## Teaser

**Summary:** Behavioral abnormalities are associated with a deficit in dopaminergic signaling in the prefrontal cortex of *Col6a1*^–/–^ mice, and patients bearing COL6 mutations show defects in attentional control abilities.

## INTRODUCTION

The extracellular matrix (ECM) displays a restricted and distinctive organization in the central nervous system (CNS), and the proper expression and dynamically regulated deposition of ECM components play critical roles in CNS development, maturation of neural circuits and adult neuroplasticity (Hubert et al., 2009). Collagen VI (COL6) is a major ECM component with emerging relevance for the nervous system (Gregorio et al., 2018), besides its established roles in skeletal muscles and connective tissues (Grumati et al., 2010; Cescon et al., 2015, 2018). Mutations of the *COL6A1-COL6A6* genes, encoding COL6 subunits, are known to be causative for a distinct subtype of congenital muscular dystrophies, including Bethlem myopathy (BM) and Ullrich congenital muscular dystrophy (UCMD) (Jöbsis et al., 1996; Camacho Vanegas et al., 2001; Merlini et al., 2008). In the past few years, COL6 mutations were also linked to central and peripheral neurological disorders, such as early-onset isolated dystonia (Zech et al., 2015), progressive myoclonus epilepsy syndrome (Karkheiran et al., 2013) and neuropathic itch (Martinelli-Boneschi et al., 2017). Furthermore, some polymorphisms of COL6 genes were recently identified as rare risk variants for schizophrenia and bipolar disorder (Salvoro et al., 2018).

The roles exerted by COL6 in the CNS are not yet fully understood. In the brain, COL6 is abundant in basement membranes and is found in close proximity to blood vessels and meninges (Gregorio et al., 2018; Roggendorf et al., 1988; Sievers et al., 1994). A neuroprotective effect for COL6 has been described against the toxicity of amyloid β-peptides *in vivo* (Cheng et al., 2009) and cell death induced by ultraviolet irradiation in cultured neurons (Cheng et al., 2011). In previous studies, we found that COL6 exerts critical functions in the aging CNS, as a lack of COL6 causes defective regulation of autophagy, increased susceptibility to oxidative stress and spontaneous apoptosis of neural cells both *in vitro* and in aged mouse brains (Cescon et al., 2016). However, in-depth studies aimed at ascertaining the functional consequences of COL6 deficiency in the CNS were still missing.

Here, we show that COL6-null mice displayed altered behaviors, accompanied by alterations in the related regulatory mechanism of the prefrontal cortex (PFC). Consistent with these behavioral abnormalities, whole-brain dopamine levels were dysregulated and dopaminergic gene expression in the PFC was distinctively altered in mice lacking COL6. In agreement with this, *in vitro* experiments revealed that primary meningeal COL6-null cells were less able to sustain SH-SY5Y dopaminergic differentiation than wild-type cells, supporting a direct link between COL6 deficiency and weaker dopaminergic development. Based on these results, we defined a protocol for neuropsychological tests that was administered to a set of BM and UCMD patients bearing identified COL6 mutations. The results of these tests indicated mildly compromised attention control in BM and UCMD patients, pointing towards a role for COL6 in sustaining dopamine- and PFC-regulated tasks in mice and humans.

## RESULTS

### *Col6a1*^–/–^ mice display sensorimotor gating deficits and memory/attention deficits

**Fig. 1.**
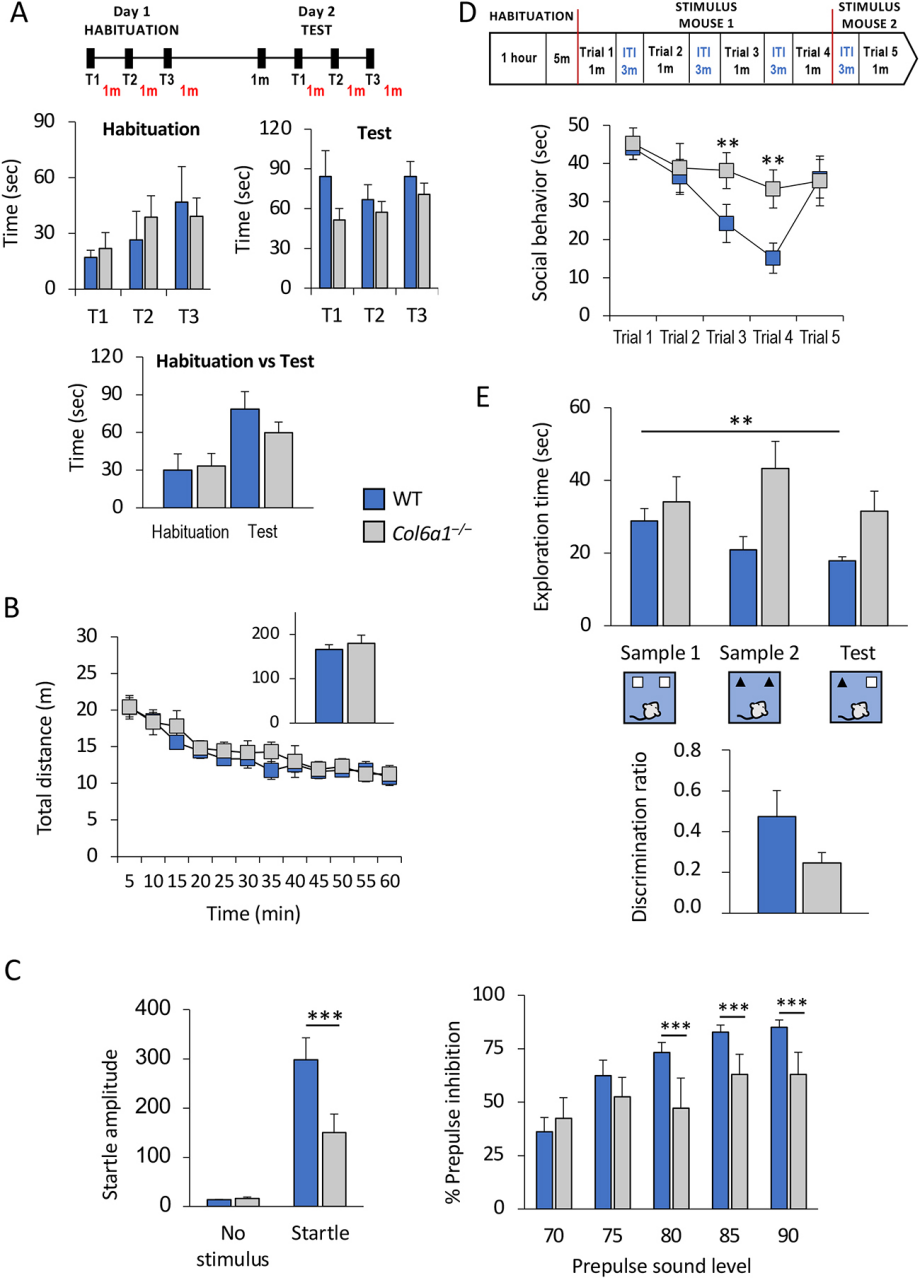
***Col6a1*^–/–^ mice display alterations in sensorimotor gating, social behavior and working memory.** Results of different behavioral analyses conducted in 3- to 6-month-old wild-type and *Col6a1*^–/–^ mice. (A) Latency to fall in each trial of the rotarod test during the habituation and the test phases, according to the protocol shown in the top panel (minute, m; trial, T). The average value among the three trials for each phase is reported in the bottom panel. (B) Ambulatory distance displayed during exposure to an empty open-field arena (inset shows the total distance traveled in 60 min). (C) Left: startle response amplitude (arbitrary units) displayed by animals following the presentation of no stimulus or a 120 dB acoustic stimulus (Startle). Right: percentage of inhibition of the acoustic startle response displayed by the same mice after the presentation of a 70, 75, 80, 85 and 90 dB prepulse stimulus. (D) Time spent in the investigation of the same unfamiliar mouse during each of four successive 1-min trials and towards the stimulus mouse (new unfamiliar mouse) presented in the fifth trial, according to the protocol shown in the top panel (inter-trial interval, ITI). (E) Total exploration time in each phase of the temporal order object recognition task. Lower panel: discrimination ratio displayed by mice during the test phase (sample 3). Although no significant differences were detected in general locomotor activity, as measured by rotarod (A), nor in an open-field arena (B), *Col6a1*^–/–^ mice showed reduced prepulse inhibition and startle amplitude reaction (C), loss of preference between familiar and non-familiar conspecific animals (D), and defective temporal order object recognition (E). Error bars indicate s.e.m. **P<0.01; ***P<0.001; two-way ANOVA with Newman–Keuls post hoc test; WT, *n*=11; *Col6a1*^–/–^, *n*=9.

We first assessed whether *Col6a1*^–/–^ mice might display any behavioral abnormality related to neurodevelopmental defects. To this end, we carried out a range of behavioral studies on adult mice that were 3 to 6 months old, and on the same mice 6 months later, long before the onset of the aging-related neurological features previously found in 20-month-old *Col6a1*^–/–^ mice (Cescon et al., 2016). *Col6a1*^–/–^ animals did not show any significant change in motor coordination function when subjected to the rotarod test (Fig. 1A), nor in exploratory behavior assessed in an open-field test (Fig. 1B; Fig. S1), indicating that the general behavioral features of adult *Col6a1*^–/–^ mice were not affected by major locomotor defects.

We next explored more specific behaviors involving CNS functioning, and in particular sensorimotor gating measures and memory functions (Managò et al., 2016; Mereu et al., 2017). Intriguingly, *Col6a1*^–/–^ mice showed a significant impairment in prepulse inhibition (PPI) (Fig. 1C), as well as in startle amplitude responses (Fig. 1C). In addition, and differently from wild-type mice, when tested in a habituation/dishabituation social task, *Col6a1*^–/–^ mice did not display the characteristic decline in the time spent investigating a same-sex conspecific when becoming familiar (Fig. 1D), a pattern consistent with a social amnesia phenotype (Ferguson et al., 2000; Huang et al., 2014). We then checked whether this phenotype was displayed only for socially relevant stimuli or was linked to a more generalized impairment. In agreement with the latter case, *Col6a1*^–/–^ animals did not show the expected decline in the time spent investigating inanimate objects following consecutive exposures (Fig. 1E). These findings suggested that COL6 deficiency results in memory and attentional deficits before aging. When tested at an older age (12 months), *Col6a1*^–/–^ mice displayed increased time freezing in an open field, enduring startle and sensorimotor gating deficits, and a similar social, but not object-related, amnesia (all relevant results are collected in Fig. S2). Taken together, these data highlight that at relatively young ages, COL6 disruption impacts CNS-relevant behavioral phenotypes.

### Decreased amounts of dopamine and serotonin are found in *Col6a1*^–/–^ mouse brains

**Fig. 2.**
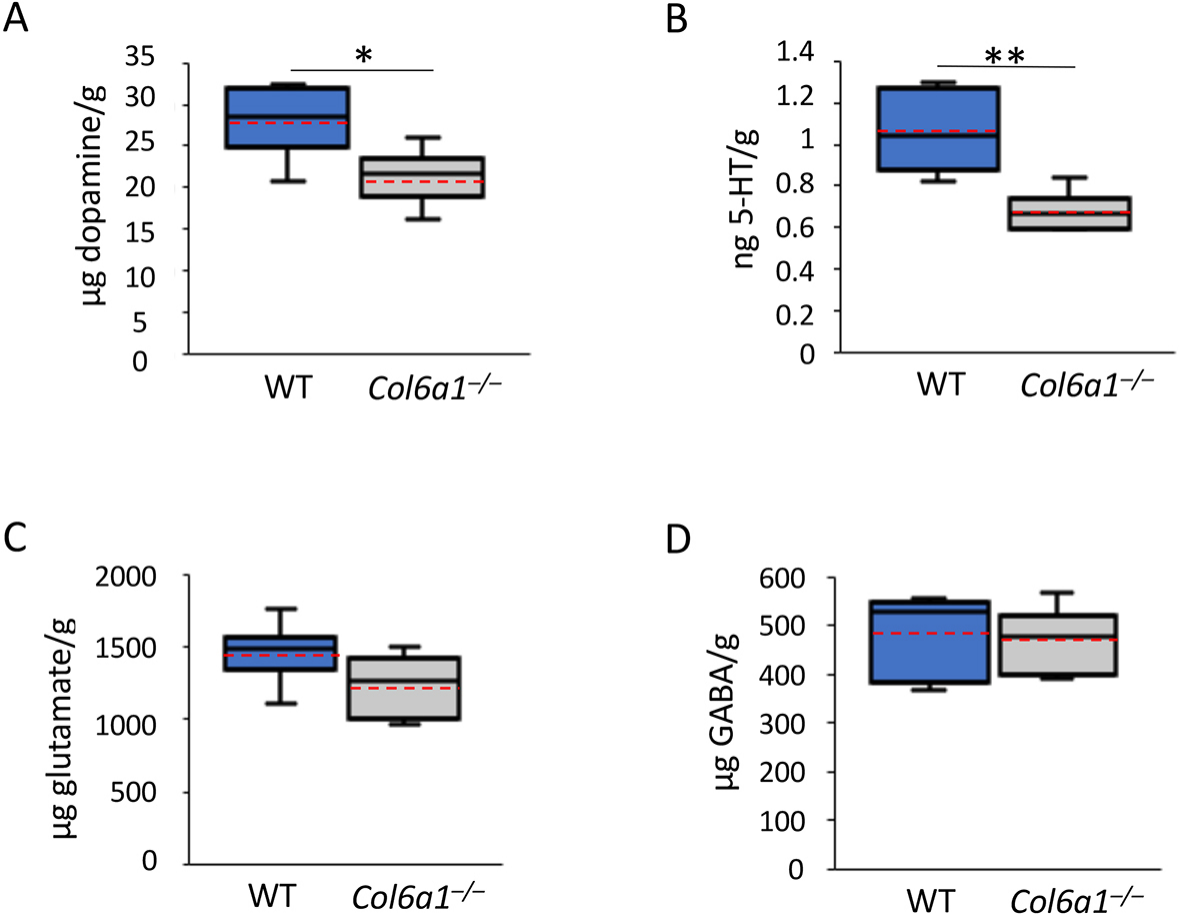
**LC-MS/MS analysis of wild-type and *Col6a1*^–/–^ mouse brains reveals altered neurotransmitter levels.** Levels of dopamine (A) and serotonin (5-HT) (B) in *Col6a1*^–/–^ brains are reduced compared with their levels in WT brains. The amount of the main excitatory (glutamate) (C) and inhibitory (GABA) (D) neurotransmitters does not change between WT and *Col6a1*^–/–^ brains. Values represent nanograms or micrograms of neurotransmitter per gram of brain tissue. The box plots show the 25-75th percentiles, solid black lines in the middle indicate the median, red dotted lines indicate mean values, and whiskers show the minimum and maximum values. *P<0.05; **P<0.01; unpaired two-tailed Student's *t-*test; *n*=6 mice for each genotype).

Prompted by the behavioral findings described above, we assessed whether any alteration in brain neurochemistry could be detected in adult *Col6a1*^–/–^ mice. Thus, we investigated some major neurotransmitters and measured the amounts of dopamine, serotonin, glutamate and γ-amino butyric acid (GABA) using liquid chromatography coupled with tandem mass spectrometry (LC-MS/MS). Remarkably, the levels of the two monoamines dopamine and serotonin were significantly decreased in the brains of *Col6a1*^–/–^ mice compared with wild-type mice (Fig. 2A,B). In contrast, the levels of glutamate and GABA did not differ between the two genotypes (Fig. 2C,D). Intriguingly, abnormal dopamine signaling has been linked with PPI, startle and memory deficits in mouse models of CNS dysfunctions, and it is considered a hallmark of different psychiatric conditions (Brisch et al., 2014; Managò et al., 2016; Mereu et al., 2017).

### *Col6a1*^–/–^ mice show dopamine signaling alterations in the medial PFC

**Fig. 3.**
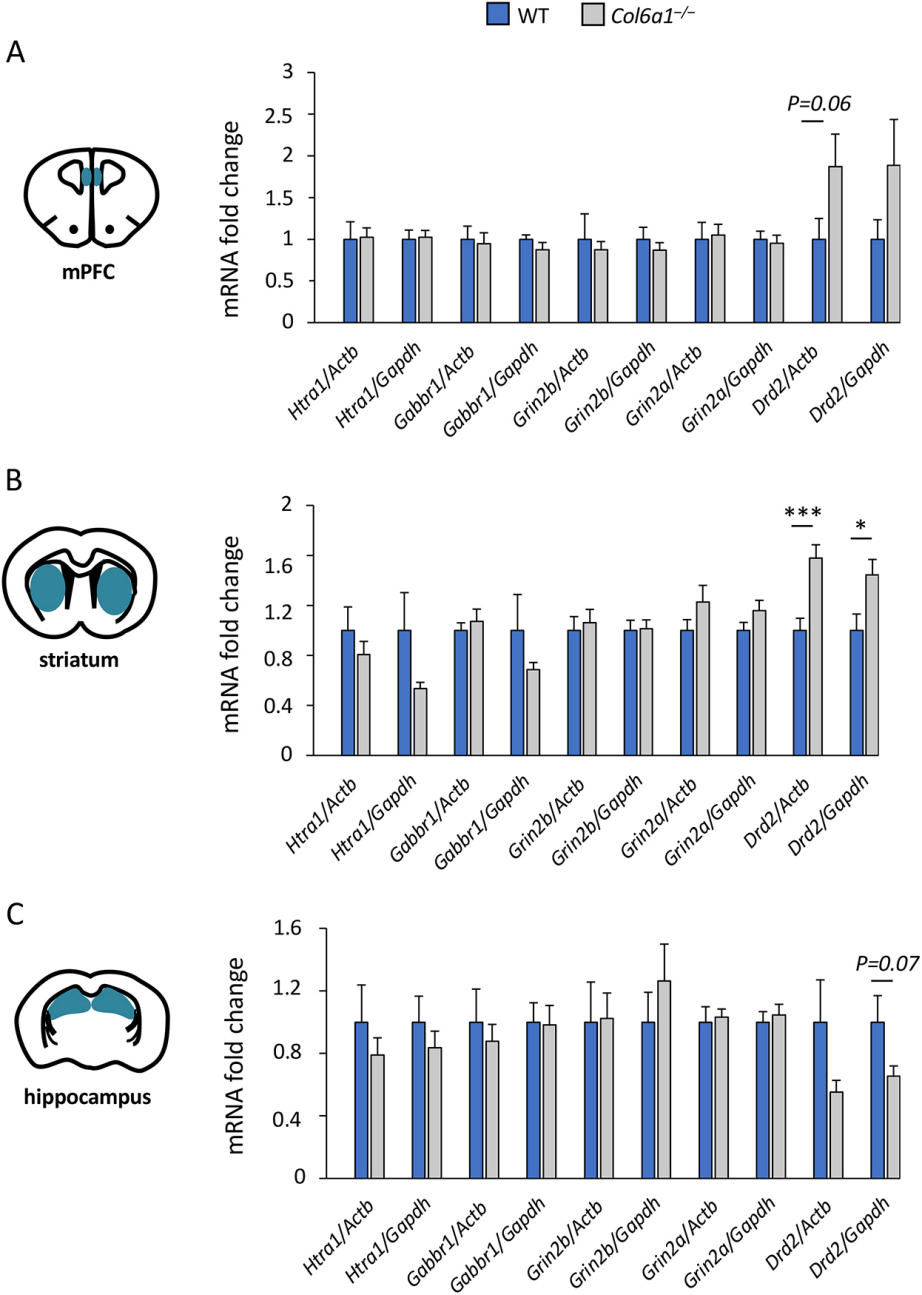
***Drd2* expression is altered in *Col6a1*^–/–^ mouse brains.** (A-C) qRT-PCR analysis of *Htra1*, *Gabbr1*, *Grin2a*, *Grin2b* and *Drd2* mRNA levels in the medial PFC (mPFC) (A), striatum (B) and hippocampus (C) of WT and *Col6a1*^–/–^ mice. Data normalized to both *Gapdh* and *Actb* (β-actin) are shown. Error bars indicate s.e.m. *P<0.05; ***P<0.001; unpaired two-tailed Student's *t-*test; *n*=12 mice for each genotype.

To understand the impact of COL6 deficiency in the brain monoaminergic systems, we analyzed the expression of genes coding for serotonin receptor 1A (*Htra1*), GABA receptor 1 (*Gabbr1*), glutamate receptors (*Grin2a* and *Grin2b*) and dopamine D2 receptor (*Drd2*) in different brain regions. In particular, we focused on the medial PFC, striatum and hippocampus, as these are the main brain regions known to contribute to the startle response and to social and memory abilities, also being involved in the dopamine hypothesis of psychiatric disorders (Winterer and Weinberger, 2004; Wan et al., 2012; Sigurdsson and Duvarci, 2016). The expression of *Drd2* was the most perturbed in all three brain regions. In particular, *Drd2* mRNA levels were significantly increased in the medial PFC and striatum of *Col6a1*^–/–^ mice and showed a slight decrease in the *Col6a1*^–/–^ hippocampus. In contrast, no alterations were detected in the expression of the genes encoding other neurotransmitter receptors (Fig. 3A-C). Notably, alterations in D2 dopamine receptor expression and signaling were consistently reported in psychiatric disorders and are relevant for pharmacological treatments (Ralph-Williams et al., 2002; Papaleo et al., 2012a; Ripke et al., 2013).

**Fig. 4.**
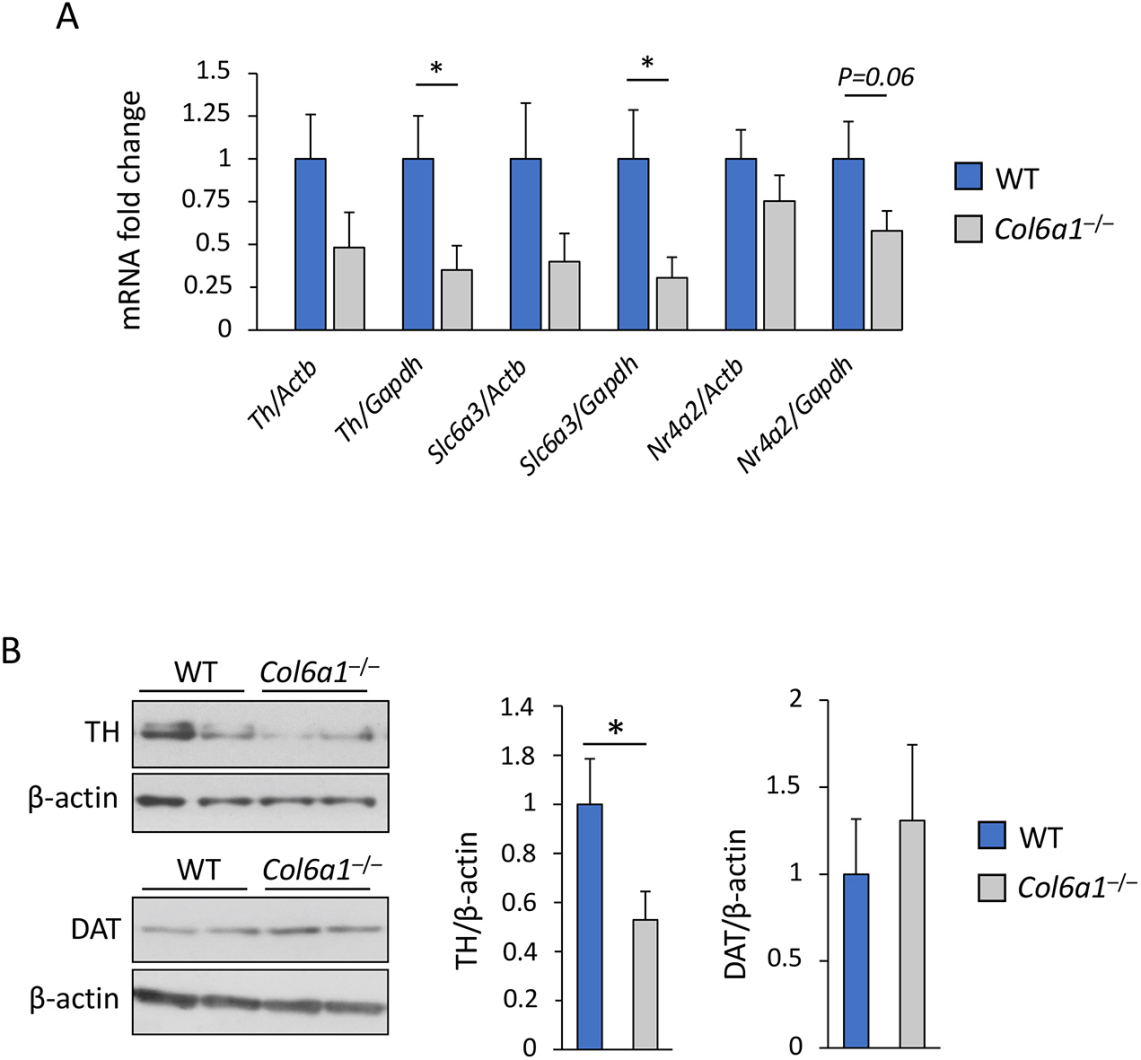
**Dopaminergic signaling is impaired in the medial PFC of *Col6a1*^–/–^ mice.** (A) qRT-PCR analysis for *Th*, *Slc6a3* and *Nr4a2* mRNA levels in the medial PFC of wild-type and *Col6a1*^–/–^ mice. *P<0.05; unpaired two-tailed Student's *t-*test; WT, *n*=11; *Col6a1*^–/–^, *n*=12. (B) Representative western blot (left) and densitometric analysis (right) of TH and DAT in the medial PFC of WT and *Col6a1*^–/–^ mice. *P<0.05; unpaired two-tailed Student's *t-*test; WT, *n*=9; *Col6a1*^–/–^ *n*=8. Error bars indicate s.e.m.

As the above data supported the concept that the altered behavioral features of COL6-null mice might be associated with altered dopaminergic transmission, we further examined dopaminergic signaling in the brains of *Col6a1*^–/–^ mice. We analyzed the expression of different key players in dopaminergic transmission, namely, the tyrosine hydroxylase enzyme TH (encoded by *Th*), involved in dopamine synthesis; the dopamine transporter DAT (*Slc6a3*), critical for synaptic dopamine reuptake; and the nuclear receptor 4A2 (*Nr4a2*), involved in the maintenance of dopaminergic neurons. Interestingly, the levels of *Th*, *Slc6a3* and *Nr4a2* transcripts were significantly decreased in the *Col6a1*^–/–^ medial PFC (Fig. 4A), but not in the other two analyzed regions (Fig. S3A,B). The decreased *Th* transcript levels were paralleled by significantly lower TH protein levels (Fig. 4B). A trend for decreased TH protein levels was also displayed in the striatum and hippocampus, albeit not significant (Fig. S3A,B). Taken together, these findings indicate that *Col6a1*^–/–^ mice display neurochemical defects impacting dopamine signaling, mainly in the medial PFC.

### *Col6a1*^–/–^ meningeal cells are unable to sustain *in vitro* dopaminergic differentiation

**Fig. 5.**
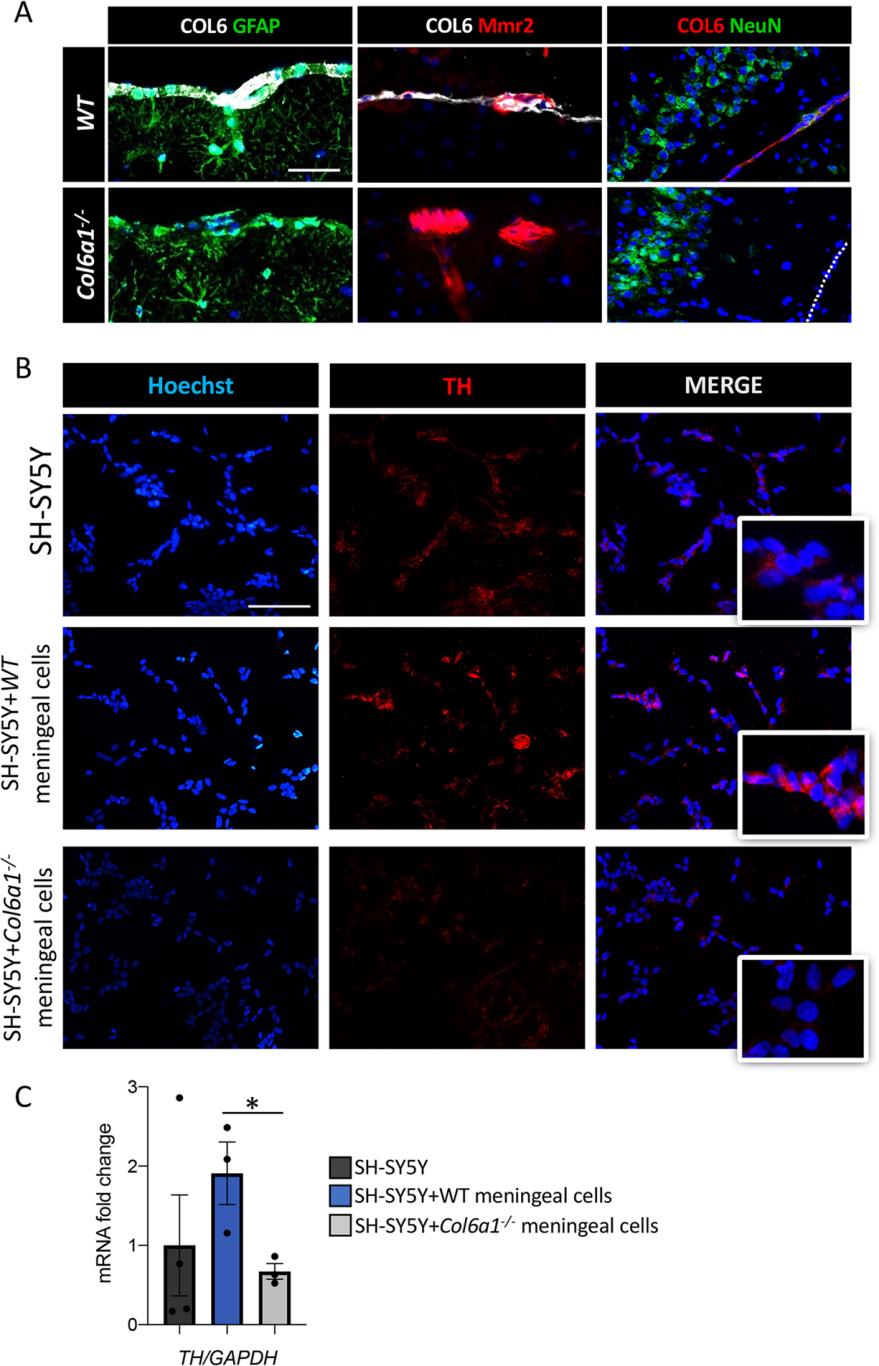
**Meningeal cells from *Col6a1*^–/–^ mice are not able to induce dopaminergic differentiation of SH-SY5Y cells.** (A) Immunohistochemistry for COL6 on WT and *Col6a1*^–/–^ brain tissues. COL6 labeling is abundant at the level of the meninges, leptomeninges and the adventitia of large blood vessels, as indicated by GFAP staining (left panels), which marks astrocytic endfeet at the pial surface, and Mmr2 staining (middle panels), which marks endothelial cells of leptomeningeal vessels. COL6 is not detectable in the brain parenchyma or in neurons, stained by the anti-NeuN antibody (right panels). Scale bars: 50 µm. (B) Representative fluorescence microscopy images of SH-SY5Y cells cultured alone (top panels) or co-cultured with WT (middle panels) or *Col6a1*^–/–^ (bottom panels) meningeal cells, stained for nuclei (Hoechst 33528, blue, left panels), TH (red, middle panels) and merged (right panels). The insets show higher magnifications of merged images. Increased TH signal was observed when SH-SY5Y cells were co-cultured with WT meningeal cells, but not with *Col6a1*^–/–^ meningeal cells. Three experimental replicates were performed. Scale bar: 100 µm. (C) qRT-PCR analysis for *TH* transcript levels in SHSY5Y cells in the absence of meningeal cells and after co-culture with WT or *Col6a1*^–/–^ meningeal cells. Error bars indicate s.e.m. *P<0.05; unpaired two-tailed Student's *t-*test; *n*=3 different experiments.

We next investigated the cellular mechanism by which COL6 influences the dopaminergic system. Within the CNS, COL6 was reported to be a major component of the ECM deposited in the meninges and blood vessels (Roggendorf et al., 1988; Sievers et al., 1994; Cescon et al., 2016). Accordingly, we detected strong COL6 immunoreactivity in the adventitia of large blood vessels and in meninges of 3- to 6-month-old wild-type mouse brains (Fig. 5A). Factors released from meninges can influence neuronal differentiation during development (Siegenthaler et al., 2009; Suter et al., 2017) and sustain dopaminergic differentiation (Hayashi et al., 2008; Schwartz et al., 2012; Somaa et al., 2015). Based on this, we hypothesized that lack of COL6 might impact meningeal cells and, in turn, their ability to sustain dopaminergic differentiation. To test this hypothesis, we established an *in vitro* co-culture system that consisted of the neuroblastoma-derived human cell line SH-SY5Y, which displays catecholaminergic neuronal properties (Xicoy et al., 2017), and primary meningeal cells derived from wild-type and *Col6a1*^–/–^ pups, and compared the properties of these co-cultures with those of SH-SY5Y cells cultured alone in the absence of other cell types. As expected, primary meningeal cells were characterized by the presence of fibroblast markers and by the absence of astrocyte endothelial markers (Fig. S4). Deposition of COL6 by wild-type meningeal cells was also confirmed (Fig. S4). Immunofluorescence showed increased TH labeling when SH-SY5Y were cultured in the presence of wild-type meningeal cells, but not when they were cultured in the presence of COL6-null meningeal cells (Fig. 5B). In agreement with this, *Th* transcript levels were significantly increased in the presence of wild-type meningeal cells, whereas no difference was found between SH-SY5Y cells cultured alone and those co-cultured with *Col6a1*^–/–^ meningeal cells (Fig. 5C). Mechanistically, these results imply that the altered dopaminergic signaling of COL6-null brains relies on the inability of meningeal cells to correctly regulate dopaminergic neuron development and homeostasis.

### Neuropsychological studies reveal subtle impairments in attention and executive function domains in patients carrying COL6 mutations

Altered PFC functions in humans might result in deficits in cognitive domains related to attention and executive functions (Dimitrov et al., 2003; Schwartz et al., 2012; Sannino et al., 2017; Leggio et al., 2021). To investigate the impact of COL6 deficiency in the CNS of subjects carrying mutations of the *COL6A1*, *COL6A2* or *COL6A3* genes, a battery of neuropsychological tests was administered to a cohort of eight patients. The main clinical and genetic features of recruited patients are summarized in Table S2.

**Fig. 6.**
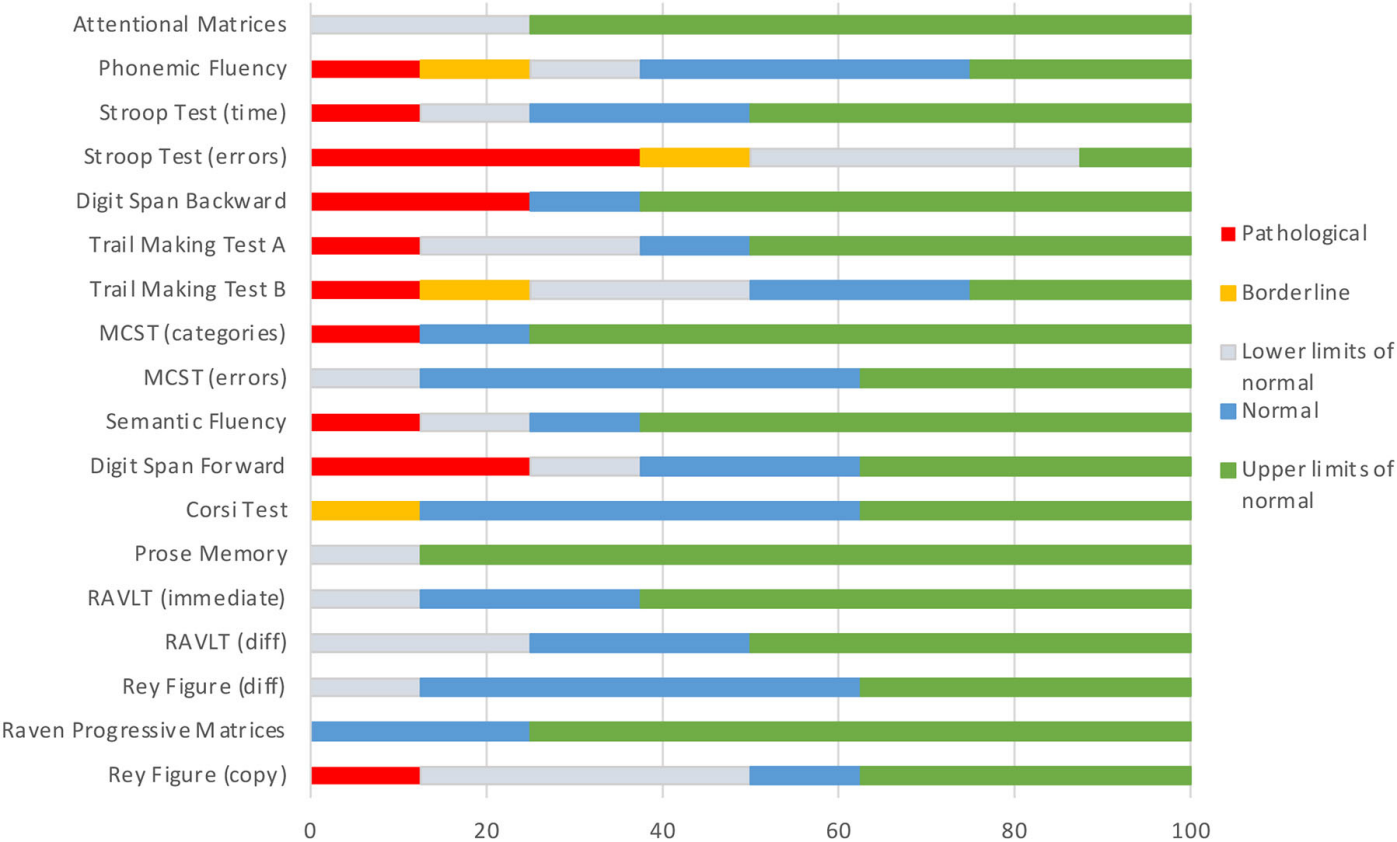
**Neuropsychological assessment in patients affected by COL6-related disorders.** Percentage of patients for each score class in each test according to the equivalent scores, reported in Table 1. Executive functions are the most impaired. In the Stroop Test (errors), 40% of patients had a pathological score, 10% a borderline score and 40% a score at the lower limits of normal. Modified Wisconsin Card Sorting Tests, MCST; Rey Auditory Verbal Learning Test, RAVLT.

Patient scores were all within normal ranges in the Mini-Mental State Examination (MMSE), in which four patients had a score of 28/30, one patient a score of 29/30 and three patients a score of 30/30. The equivalent scores of different neuropsychological tests, encompassing various classes of cognitive domains (attention and executive functions, language, memory, abstract reasoning and visuospatial skills), were evaluated for each patient (see Table 1). Scores were divided into five classes: upper limits of normal, normal, lower limits of normal, borderline and pathological. The percentage of patients in each class for each cognitive domain was calculated (Fig. 6).

**Table 1.**
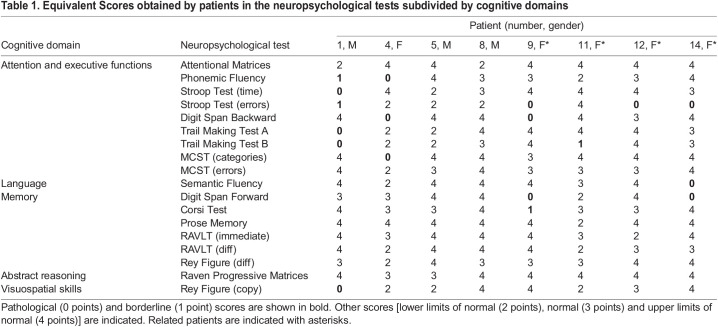
Equivalent Scores obtained by patients in the neuropsychological tests subdivided by cognitive domains

Two patients (patient 5, intermediate UCMD, and patient 8, BM) showed normal performances in all tasks. The domain related to attention and executive functions was affected in all the other patients (Table 1). Patient 1 (UCMD) displayed a pathological 0 score in the Stroop Test Time, measuring the time needed to complete the task. Patients 9, 12 and 14 (all BM) scored 0 and patient 1 (UCMD) scored 1 in the Stroop Test errors. At the Digit Span Backward task, patients 4 (intermediate UCMD) and 9 (BM) scored 0. Patient 1 (UCMD) had a pathological score of 0 in both the Trail Making Test (TMT) A and B, whereas patient 11 (BM) had a borderline performance in TMT B (equivalent score of 1). In the Modified Card Sorting Test (MCST), only patient 4 (intermediate UCMD) had a pathological 0 score. Patient 14 (BM) scored below the cut-off in the language domain. Patients 9 (BM) and 14 (BM) had a deficit in verbal short-term memory (Digit Span Forward Test). Patient 9 (BM) also had a borderline performance in spatial short-term memory (Corsi test). In all tests assessing long-term memory and learning [prose memory, Rey Auditory Verbal Learning Test (RAVLT) and Rey figure recall], all patients obtained a performance within normal range. Abstract reasoning scores (Raven Progressive Matrices) were normal or above normal values in all patients. Patient 1 (UCMD) scored below the cut-off in the Rey–Osterrieth Complex Figure test, investigating visuospatial abilities.

## DISCUSSION

Previous studies have shown that COL6 deficiency has a remarkable impact on key cellular processes, such as apoptosis, autophagy and oxidative damage, which are affected in both the brain and skeletal muscles, pointing towards a cytoprotective role of COL6 during physiological aging (Cescon et al., 2016; Capitanio et al., 2017). In the present study, we investigated whether COL6 deficiency leads to functional CNS deficits, such as memory and sensorimotor gating impairments, by exploiting a battery of specific tests in *Col6a1*^–/–^ mice, thus avoiding potential biases attributable to the myopathic phenotype (Irwin et al., 2003; Bonaldo et al., 1998). Locomotor-dependent tests, such as rotarod and free exploratory activity in an open-field arena, did not show any significant difference between young (3- to 6-month-old) wild-type and *Col6a1*^–/–^ mice, confirming that the myopathic phenotype does not affect the general locomotor capabilities of COL6-deficient mice, in agreement with previous work (Chen et al., 2014).

Conversely, behavioral tests assessing sensorimotor gating, such as PPI and memory for social or inanimate cues, highlighted marked differences between wild-type and *Col6a1*^–/–^ mice. PPI is the inhibition of a startle response to an abrupt environmental/acoustic stimulus, when this is preceded by a prepulse of lower intensity (Ison et al., 1973). Considered to be a well-grounded neurophysiological measurement in translational research, with major relevance for schizophrenia (Powell et al., 2009; Papaleo et al., 2012b; Braff et al., 2005), PPI corresponds to the inhibition of unwanted repetitive and distracting stimuli, controlled by complementary circuitry distributed in the PFC, striatum, hippocampal and limbic brain regions, and modulated by several molecular mediators including dopamine, which acts through D2 receptors (Swerdlow, 2009; Powell et al., 2009). Dopaminergic neurotransmission was widely reported to be involved in PPI modulation, with PPI being disrupted by both hyperdopaminergic activity (Peng et al., 1990; Wan et al., 1996) and by cortical hypodopaminergic transmission, as witnessed by genetic and pharmacological *in vivo* models (Managò et al., 2016; Zavitsanou et al., 1999). In COL6-null animals, PPI is impaired in terms of reduced inhibition of the startle response, which is elicited by high-intensity prepulses, in line with the presence of an impaired dopamine functionality in the PFC. PPI in rodents and humans is also modulated by the serotonin/dopamine balance (Fletcher et al., 2001; Mann et al., 2008). Remarkably, not only dopamine levels but also serotonin levels were reduced in COL6-null brains.

Furthermore, COL6-null mice display an impaired capability to habituate their exploration towards a familiar conspecific animal or object. This phenotype relies upon a deficit in remembering that the same stimulus was already presented to the tested subject (i.e. memory deficits), or alternatively an inattentiveness to previously encountered social or object cues. Such a memory/inattentiveness phenotype has been linked to dopamine dysregulation (Dash et al., 2007). Indeed, too little and too much dopamine can impair working memory and cognitive control (Goldman-Rakic et al., 2000; Cools and D'Esposito, 2011; Floresco, 2013; Deng et al., 2015). Although hyperdopaminergic activity was widely addressed in animal studies related to working memory impairment (Zahrt et al., 1997; Leo et al., 2018), PFC-specific dopamine depletion in monkeys and administration of dopamine receptor antagonists in rats were found to lead to impaired memory retrieval in decision-making processes (Brozoski et al., 1979; Bubser and Schmidt, 1990; Seamans et al., 1998). In agreement with these latter findings, our results point to altered dopaminergic signaling in *Col6a1*^–/–^ animals, highlighted by lower dopamine levels, both in terms of neurotransmitter levels in whole-brain homogenates and of TH expression, which are more prominent in the PFC region. In parallel to TH downregulation and dopamine decline, *Drd2* mRNA levels were significantly increased, whereas the *Slc6a3* gene was found to be downregulated in the PFC of *Col6a1*^–/–^ mice. Indeed, compensatory mechanisms are expected to be established in order to enhance dopamine signaling, including reduced dopamine re-uptake by transporter downregulation and increased levels of D2-type dopamine receptors. Similar compensations were observed in Parkinson's disease and in other models of dopamine depletion (Blesa et al., 2017; Sun et al., 2013). In schizophrenia patients and models, low dopamine activity in the PFC was assumed to cause cognitive symptoms, whereas increased D2 dopamine receptor densities were detectable (Seeman and Niznik, 1990; Brisch et al., 2014).

In parallel, patients affected by COL6-related myopathies were administered an ad hoc neuropsychological protocol exploring different classes of cognitive domains, including attention and executive functions, language, memory, abstract reasoning and visuospatial functions. Although no overt intellectual disability was reported for BM or UCMD patients (Nadeau et al., 2009), to our knowledge no literature studies have been conducted to assess patients' abilities in fine functional tasks. The neuropsychological assessment performed in this cohort of BM and UCMD patients confirmed that most cognitive abilities were normal. Remarkably, all domains were preserved in the evaluated patients, except for executive functions, which were affected in 6 out of 8 patients. In particular, the Stroop Test was abnormal in four and borderline in three patients. Interestingly, the average score for the Stroop Test did not differ among UCMD and BM patients, and no specific correlation with age was found. The Stroop Test investigates prefrontal functions and is frequently abnormal in patients with frontal lesions (Bench et al., 1993). This test measures the ability to inhibit distractive interfering information while processing other information that has a fixed priority (Stroop, 1935; Sarason et al., 1995). Of note, previous studies showed that D2 receptor agonists, such as bromocriptine, were able to induce a higher inhibition of irrelevant information in the Stroop Test in healthy subjects (Roesch-Ely et al., 2005; Kimberg et al., 1997), in line with other works showing hypodopaminergic activity in clinical cohorts with impaired executive functions. Relevant disease conditions characterized by hypodopaminergic neurotransmission, including Parkinson's disease (Costa et al., 2014) and attention deficit/hyperactivity disorder (Tannock et al., 2006), show worse Stroop Test performances than controls. Our data in the evaluated cohort of BM/UCMD patients suggest tasks tapping executive functions to be particularly sensitive to COL6 deficiency, whereas equally demanding tasks, such as those evaluating abstract reasoning, memory and visuospatial skills, are unaffected. The good performance at the MMSE in all patients underscores how their deficits have a limited influence on their general cognitive, daily life activities. Given the low prevalence of COL6-related myopathies, the relatively small sample size of our patient cohort, which also includes some related individuals, does not allow drawing definite broad-spectrum conclusions; nevertheless, the results obtained with the administered battery of psychometric tests unequivocally point at subtle, PFC-dependent, functional impairment, consistent with hypodopaminergic activity. Of note, although studies performed in the *Col6a1*^–/–^ model are related to a context in which COL6 is completely ablated (Cescon et al., 2015), we cannot infer that mutations in different COL6 genes have equivalent effects in a neuropsychological context.

ECM remodeling has been associated with axonal guidance functions, structural stabilization of myelinated fiber tracts, synaptic plasticity and stem-cell maintenance, as well as organization of the CNS basement membrane, with major implications for the neurovascular unit and the blood–brain barrier (Zimmermann and Dours-Zimmermann, 2008; Berretta, 2012; Ferrer-Ferrer and Dityatev, 2018). Indeed, different ECM proteins are associated with psychiatric diseases and cognitive impairment in humans, including reelin, relevant for schizophrenia and autism (Fatemi, 2001; Ovadia and Shifman, 2011; Enwright et al., 2016); chondroitin sulfate proteoglycans, dysregulated in schizophrenic patients and critical for correct behavioral pattern in mice (Berretta, 2012; Yoshioka et al., 2017); matrix metalloproteinases, involved in synaptic plasticity (Chopra et al., 2015; Lepeta and Kaczmarek, 2015); and tenascins, linked to psychiatric phenotypes in patients and mice (Garcion et al., 2001; Kawakami and Matsumoto, 2011). In this context, it is interesting to mention that reelin heterozygous knockout (*Reln*^+/–^) mice display lower levels of dopamine, lower TH expression, defective sensorimotor gating and fine cognitive alterations (Brigman et al., 2006; Barr et al., 2008; Michetti et al., 2014), and that in the PFC of *Col6a1*^–/–^ mice, we detected a decrease of *Reln* expression by nearly 50% (Fig. S5). Of note, one recent study focusing on a cohort of schizophrenia and bipolar disorder patients within a closed population sample with high prevalence of psychiatric disorders identified rare COL6 sequence variants shared by patients from multiple families, but not by unaffected relatives or controls (Salvoro et al., 2018). This strongly suggests the need for further studies aimed at investigating whether COL6 might exert an as-of-yet underestimated role in neurodevelopment and the etiopathology of psychotic disorders.

Concerning the underlying mechanisms linking COL6 deficiency with dopaminergic dysregulation, the most abundant COL6 deposition in CNS is found within large brain vessels and meninges. Meningeal cells are fibroblast-like cells that organize the pial ECM, thereby supporting and protecting the brain parenchyma. Several studies highlighted the role of the meninges in corticogenesis and in regulating CNS homeostasis, owing to their ability to act as a stem-cell niche and regulate the secretion of trophic factors (Decimo et al., 2012; Siegenthaler and Pleasure, 2011). In particular, meningeal cues are essential in sustaining dopaminergic differentiation (Hayashi et al., 2008; Xicoy et al., 2017) and promote the survival of dopaminergic precursors in cell replacement therapies in animal models of Parkinson's disease (Somaa et al., 2015; Torres et al., 2007; Bye et al., 2012). Our data indicate that *Col6a1*^–/–^ meningeal cells have a reduced capability in promoting dopaminergic differentiation of neuronal cells, supporting the concept that COL6 proteins secreted by meningeal cells sustain the proper function of dopaminergic neurons.

In conclusion, our studies, by describing for the first time behavioral abnormalities associated with a distinctive deficit in dopaminergic signaling in the PFC in *Col6a1*^–/–^ mice and defects in attentional control abilities in patients bearing COL6 mutations, shed new light on the relevance of COL6 in the CNS and highlight a critical role for COL6 in sustaining dopamine- and PFC-regulated tasks in mice and humans. Furthermore, they strengthen the emerging view that defects in specific ECM components play a role in cognitive impairment and in the etiopathology of neurodevelopmental disorders.

## MATERIALS AND METHODS

### Mice

All experiments were carried out in 3- to 6-month-old wild-type and *Col6a1^–/–^* male mice from the C57BL/6N background (Cescon et al., 2018). Animal procedures were approved by the Ethics Committee of the University of Padova and authorized by the Italian Ministry of Health. Two to four animals were housed per cage in a climate-controlled animal facility and maintained on a 12 h light/dark cycle with free access to food and water. Whole brains or specific regions (PFC, striatum and hippocampus) were dissected and rapidly frozen in liquid nitrogen for protein, RNA or neurotransmitter extraction.

### Behavioral studies

The different tests were performed on mice at 3 to 6 months of age, and again on the same sets of mice 6 months later. Animals had multiple days of resting time between tests that were performed in the same order in which they are presented in this section. The order of tests was the same for each mouse.

#### Prepulse inhibition

Prepulse inhibition (PPI) was measured using four SR-LAB System (San Diego Instruments) as described (Mereu et al., 2017; Papaleo et al., 2008). PPI test sessions began by placing the mouse in the Plexiglass holding cylinder (5 cm diameter) for a 5-min acclimation period. After acclimation, each subject received seven trial types across six blocks of trials, for a total of 42 trials. Trial types were presented randomly within each block. The interval between trials was 10-20 s. One trial type measured the response to no stimulus (baseline movement), and another one presented the startle stimulus alone (startle), represented by a 40 ms, 120 dB sound. The other five were acoustic prepulse plus acoustic startle stimulus trials. Prepulse tones were 20 ms at 70, 75, 80, 85 and 90 dB, presented 100 ms before the startle stimulus (120 dB). The maximum startle amplitude was the dependent variable. A background level of 70 dB white noise was maintained over the duration of the test session.

#### Locomotor activity

The experimental apparatus consisted of four open-field arenas (42×42×30 cm), illuminated by overhead white lighting (25±5 lux). To quantify exploratory and locomotor activities a video tracking system (ANY-maze, Stoelting) was used during 1 h of the test. In order to evaluate any possible motor alterations (e.g. hyperlocomotion), we evaluated the total distance traveled (m), percentage of time in the internal zone, average speed (m/s) and time immobile or freezing (s) (Mereu et al., 2017).

#### Social habituation/dishabituation

Social habituation/dishabituation was performed to evaluate social memory as described (Ferguson et al., 2000; Huang et al., 2014). The apparatus consisted of a plastic cage (Tecniplast, 35.5×23.5×19 cm) with shaved wood bedding, which was lightly illuminated (5±1 lux) and recorded using a webcam (Logitech VC-B25). One hour before the test, each mouse was placed into the testing cage in a room adjacent to the testing room. No previous single-housing manipulation was adopted to avoid any generation of home-cage territory and aggressive behavior. Five minutes before the experiment, the cage containing the test mouse was gently moved in the testing room. The test began when the first stimulus mouse (S1), matched for sex and age, was introduced to the cage for the first 1 min of interaction. At the end of the 1-min trial, the stimulus animal was removed and returned into the individual holding cage. We introduced the S1 mouse for four trials with 3-min intervals between trials. The last trial was a ‘dishabituation’ test, in which a new unfamiliar stimulus mouse (S2), matched for sex and age, was presented to the test mouse. Videos of behaviors were recorded and subsequently scored offline.

#### Rotarod

The rotarod test was used to investigate motor coordination and balance as previously described (Crawley, 2007), and lasted 2 days. On day 1, mice were trained to the rotation of the rod under a constant speed of 4 rpm for three trials, with a 1-min pause between each trial. The trial ended when mice fell off the rod or until they were able to stay on the rod for 5 min. On day 2, mice were tested for three trials with a 1-min interval between trials. The test started when the mice were placed on the rod under rotation at a constant 4 rpm speed for 1 min. Then, the accelerating program was launched, and the trial ended when mice fell off the rod or until they were able to stay on the rod for 5 min. Their time spent on the rod was manually measured.

#### Temporal order object recognition test

Mice were tested in an experimental apparatus consisting of an opaque open field box (42×42×30 cm) with even, overhead white lighting (25±5 lux). Each session was recorded using an overhead camera (ANY-maze, Stoelting). Each mouse was monitored for its locomotor activity in the empty open-field boxes for 1 h. The next day, the ability of the subjects to differentiate between two objects presented at different intervals was assessed. The objects presented were rectangular boxes (3×3×6 cm) or two laboratory flasks (4×2×6 cm), each either black or white and too heavy for the animals to displace. The objects were placed in two corners of the open-field apparatus, 8 cm from the side walls. This task included two sample phases and one test trial. In each sample phase, the subjects were allowed to explore two copies of an identical object for a total of 5 min. Different objects were used for sample phases 1 and 2, with a 1-hr delay between the two sample phases. The test trial (5-min duration) was performed 3 h after sample phase 2. During the test trial, a third copy of the objects from both sample phase 1 and sample phase 2 was used. The time spent exploring each object was subsequently scored from the ANY-maze videos as the number of seconds for which each subject was facing the object and was 1 cm away. If temporal order memory was intact, subjects would have spent more time exploring the object from sample 1 (i.e. the object presented less recently) compared with time spent on the object from sample 2 (i.e. the object presented more recently). Animals that failed to complete a minimum of 2 s exploration in the sample or test phases were excluded from the analysis. The discrimination ratio was calculated as the difference in time spent by each subject exploring the objects from sample phase 1 compared with time spent on the objects from sample phase 2 divided by the total time spent exploring both objects during the test period.

### Neurotransmitter analysis

Neurotransmitters in brain tissue were analyzed by LC-MS/MS. Samples (300 mg) were extracted using 1 ml methanol with 0.05% trifluoroacetic acid, added to a solution containing 100 ng benzanilide as an internal standard. After 10 min of sonication in an ice bath, samples were centrifuged at 10,000 ***g***, and the supernatant was transferred to a glass tube and dried with a gentle flow of nitrogen. The solution was reconstituted with 100 µl methanol and used for LC-MS/MS analysis by a Prostar 410 binary pump chromatograph with a Prostar Diode Array and a Triple Quadrupole MS 320 (Varian). The mass spectrometer was equipped with an electrospray ion source and compounds were ionized in positive mode. For each analyte, specific parameters and transitions were optimized using 1 ppm solutions directly infused in the electrospray ion source. The selected transition for the quantitative analysis of each analyte were as follows [indicated as parent mass-to-charge ration (m/z)>fragment m/z]: glutamic acid, 148>84; dopamine, 154>137; serotonin, 177>114; and benzanilide (internal standard), 198>105. Calibration curves for each compound were built by mixing the internal standard and solution of the respective standard. The calibration curves were *y*=46.8*x*+0.285 for GABA, *y*=329.8*x*+2.31 for glutamic acid, *y*=68.92*x*+0.325 for serotonin and *y*=203.06*x*+0.835 for dopamine. The limits of detection (LODs) and limits of quantifications (LOQs) were as follows: GABA, LOD 20 ng/g, LOQ 60 ng/g; glutamic acid, LOD 100 ng/g, LOQ 350 ng/g; and serotonin, LOD 0.05 ng/g, LOQ 0.15 ng/g.

### Isolation and culture of primary meningeal fibroblasts and co-culture with SHSY-5Y cells

Brains were dissected from mouse pups (postnatal day 4) and the meninges separated from brain parenchyma and processed as described previously (Niclou et al., 2003) to obtain primary meningeal cells. SH-SY5Y cells (American Type Culture Collection) were grown in Dulbecco's Modified Eagle Medium (DMEM)/F12 (Gibco) supplemented with 10% fetal bovine serum (Gibco) until 60% confluency was reached. Then, primary meningeal cells (fourth culture passage) from wild-type and *Col6a1^–/–^* mice were added (1500 cells/cm^2^) and co-cultured with SH-SY5Y cells for 3 days before RNA/protein purification or fixation.

### Immunofluorescence

Following fixation with paraformaldehyde, cells were permeabilized for 5 min with 0.1% Triton X-100 in PBS and blocked for 1 h with 10% goat serum in PBS. For brain slices, wild-type and *Col6a1^–/–^* brains were snap frozen in liquid nitrogen, sectioned using a cryostat (Leica CM1950), and 10 μm slices were deposited on glass slides before fixation/permeabilization in ice-cold acetone/methanol and blocking for 1 h with 10% goat serum in PBS. Samples were incubated with primary antibodies overnight at 4°C. The following day, after three PBS washes, samples were incubated with fluorophore-conjugated secondary antibodies for 1 h at room temperature, then mounted in 80% glycerol in PBS and imaged with a Leica DM5000B fluorescence microscope or a Zeiss LSM700 confocal microscope. The primary antibodies used were as follows: rabbit anti-tyrosine hydroxylase (Sigma-Aldrich, SAB2701683, 1:100); rabbit anti-vimentin (Santa Cruz Biotechnology, sc-7557, 1:100); mouse anti-α-SMA (Dako, clone 1A4, 1:100); mouse anti-GFAP (Millipore, MAB3402, 1:100); rabbit anti-laminin (kindly provided by Giorgio Maria Bressan, Department of Molecular Medicine, University of Padova, 1:100); rabbit polyclonal antisera against mouse α3(VI) collagen (kindly provided by Raimund Wagener, Center for Biochemistry, Medical Faculty, University of Cologne, 1:200); mouse monoclonal anti-NeuN (MAB377, Millipore, 1:200); hamster monoclonal anti-podoplanin (Santa Cruz Biotechnology, sc-53533, 1:100); rat anti-multimerin2 (kindly provided by Alfonso Colombatti, Centro di Riferimento Oncologico di Aviano, Aviano, Italy, 1:150). Nuclei were stained with Hoechst 33528 (Sigma-Aldrich). The secondary antibodies used were as follows: anti-mouse CY2 (115-226-062, Jackson ImmunoResearch, 1:100); anti-rabbit CY2 (111-225-144, Jackson ImmunoResearch, 1:100); anti-rabbit CY3 (111-165-144, Jackson ImmunoResearch, 1:100); anti-rat CY3 (112-165-167, Jackson ImmunoResearch, 1:100); and anti-hamster FITC (Santa Cruz Biotechnology, sc-2792m, 1:100).

### Quantitative real-time PCR

For RNA extraction, cells or brain regions (PFC, striatum, hippocampus) were lysed in TRIzol reagent (Invitrogen). Samples were processed according to the manufacturer’s instructions, and RNA was retrotranscribed using M-MLV reverse transcriptase (Invitrogen). cDNA products were analyzed by quantitative real-time PCR (qRT-PCR) with the Rotor Gene SYBR PCR Kit (QIAGEN). Primer sequences are reported in Table S1.

### Western blotting

Brain regions (PFC, striatum, hippocampus) were homogenized in lysis buffer (50 mM Tris-HCl, 150 mM NaCl, 1% IGEPAL, 0.5% sodium deoxycholate and 0.1% SDS) containing protease inhibitors (complete EDTA free, Roche) and phosphatase inhibitors (Cocktail II, Sigma-Aldrich) using a motor pestle (Kimble). Protein extracts were quantified by the BCA Protein Assay Kit (Pierce) and separated by SDS-PAGE in 4-12% polyacrylamide gels (Invitrogen). Samples were then blotted onto polyvinylidene difluoride membranes, blocked with 5% milk in 0.1% Tween 20 in TBS (TBS-T) and probed with primary antibodies overnight at 4°C. After three TBS-T washes, membranes were probed with horseradish peroxidase-conjugated secondary antibodies (Amersham Biosciences) for 1 h at room temperature. Detection was performed by chemiluminescence (Pierce). The following primary antibodies were used: rabbit anti-tyrosine hydroxylase (Sigma-Aldrich, SAB2701683, 1:1000); rat anti-DAT (Santa Cruz Biotechnology, sc-32259, 1:1000); and mouse-anti β-actin (Sigma-Aldrich, A5316, 1:1000).

### Patient selection and examination

The study was conducted in accordance with the ethical rules and guidelines issued by the local ethical committee and with the declaration of Helsinki. All clinical assessments were part of the routine clinical practice and each patient gave written informed consent to the procedures. Participants were enrolled by experienced neurologists. Patients participating in this study with genetically confirmed diagnosis of BM or UCMD were from the ERN Neuromuscular Centre of the Neurological Clinic of the University of Padova. Of the eight available patients, four were related. Age at loss of ambulation and age of start of non-invasive mechanical ventilation (NIMV) were used to classify the patients into three categories: (1) UCMD phenotype, patients who never walked or lost ambulation by 10 years of age and started NIMV by 11 years of age; (2) UCMD intermediate phenotype, patients who lost ambulation and started NIMV by 20 years of age; and (3) BM phenotype, patients who walked until adulthood and did not require NIMV.

Neurological examination included recording of abnormal gait, muscle atrophy, scoliosis, scapular winging, the occurrence and the location of contractures, and joint hyperlaxity. Muscle strength was assessed through Manual Muscle Testing in lower and upper limbs and axial muscles, according to the MRC (Medical Research Council, UK) scale. For this study, an ad hoc neuropsychological protocol was designed and administered to patients by trained psychologists. The aim of this protocol was to assess a wide range of cognitive functions to see whether selective deficits could be detected in the same cognitive domains found to be altered in mice. Thus, deficits in executive tasks were expected to contrast with otherwise spared cognitive functions. Patients therefore underwent the following tests: (1) Mini-Mental State Examination (MMSE) (Folstein et al., 1975) to assess general cognitive functioning (normal≥24/30) (Creavin et al., 2016); (2) phonemic fluency, Stroop Color and Word test (SCWT) (Scarpina and Tagini, 2017), digit cancellation test (Hatta et al., 2012), Trail Making Test A and B (Giovagnoli et al., 1996), and modified Wisconsin Card Sorting Test (WCST) (Cianchetti et al., 2005) to assess executive function and attention; (3) generating as many words as possible belonging to the same semantic category (Novelli et al., 1986) to assess an internal lexicon with semantic criteria; (4) Rey-Osterrieth complex figure (ROCF) test (Caffarra et al., 2002) to assess visuospatial abilities; (5) the digit span test (Choi et al., 2014) and the Corsi Block tapping test (Busch et al., 2005) to assess short-term memory; (6) the story recall test (de Renzi et al., 1977) and the Rey Auditory Learning Test (Carlesimo et al., 2002) to assess long-term memory; (7) Raven Progressive Matrices (RPM) (Raven, 2000) to assess abstract reasoning; and (8) Facial Expression Matching (FEM) and the Neutral Face Memory task (FaMe-N) (de Gelder et al., 2015) to assess social cognition.

For some tests, an Equivalent Score (ES) was assigned to each patient by correcting the scores using normative data for the population. The ES consists of a five-point scale that provides a solution to the problem of standardizing neuropsychological scores, free of the influences of age and education, according to the following criteria: 0, pathological; 1, borderline; 2, lower limits of normal; 3, normal; and 4, upper limits of normal (Capitani and Laiacona, 1997).

The MMSE is scored from 0 to 30 points and cannot be converted to an ES; thus, the non-converted scores were used. Mean or median values between groups were compared by two-tailed unpaired Student’s *t*-test (for normally distributed variables) or Wilcoxon–Mann–Whitney test as appropriate. The correlations between the ages of patients and functional test scores were assessed using the Spearman correlation coefficient. Significance was set at *P*<0.05.

### Statistical analysis

For mouse behavioral studies, two-way ANOVA was used to analyze the contribution of the categorization factors that were taken into account (genotype, age and repeated measures) on the outcome of the dependent variable (behavior). Newman–Keuls post hoc test was used for comparison between groups when ANOVA showed significant differences for the main factors or their interactions. The accepted value for significance was *P<*0.05. Statistical analyses were performed using Statistica 11 (StatSoft) software.

## Supplementary Material

10.1242/dmm.049481_sup1 Supplementary information
