# Supplementary material for: Collagen VI deficiency causes behavioral abnormalities and cortical dopaminergic dysfunction
Source: Dis Model Mech. 2022 Sep 21;15(9):dmm049481. doi: 10.1242/dmm.049481 (PMC9548377; doi:10.1242/dmm.049481)
Supplement: Supplementary information [file dmm-15-049481-s1.pdf]

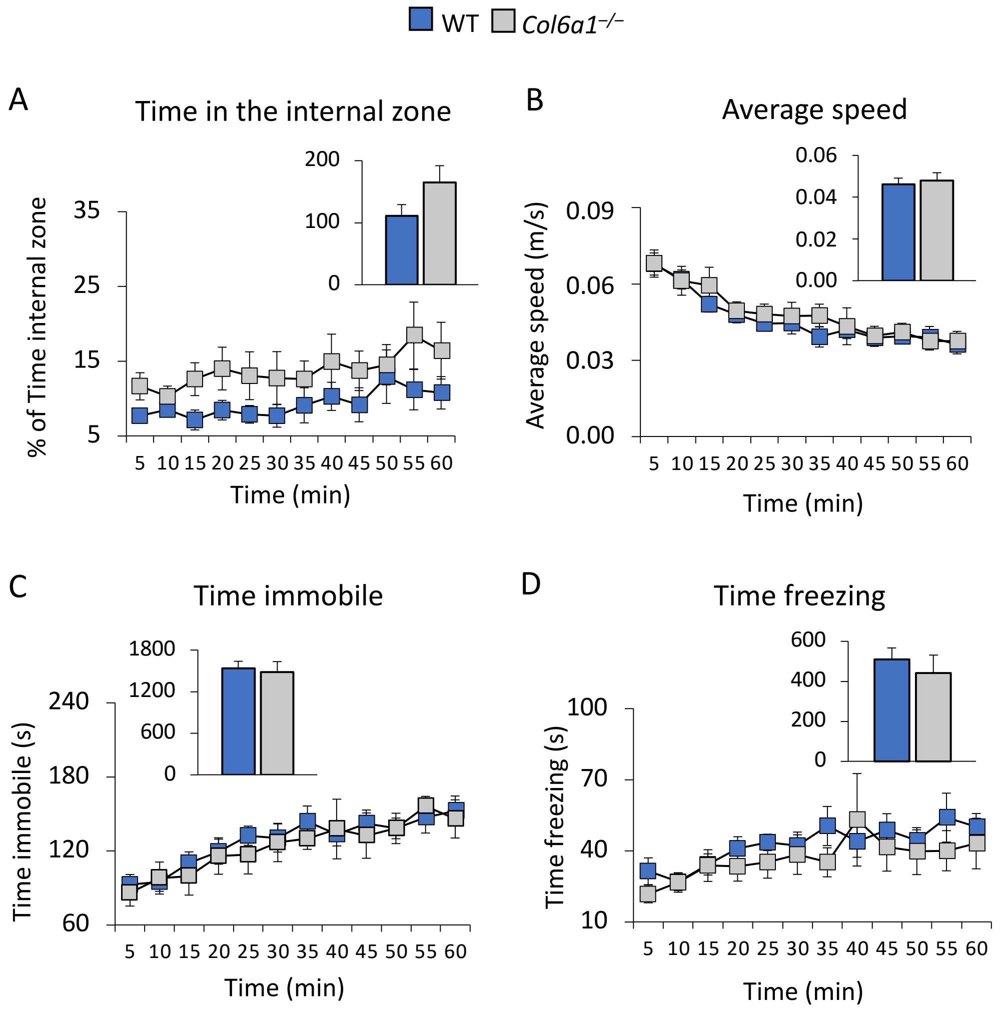

Supplementary Figure S1

Fig. S1. Additional parameters analyzed in locomotor activity open field arena test in wild-type and *Col6a1*$^{-/-}$ mice. The panels summarize the results of locomotor activity parameters in the open filed arena test performed in 3- to 6-month-old wild-type and *Col6a1*$^{-/-}$ mice. Measurements refer to (**A**) time spent in the internal zone of the arena; (**B**) average speed; (**C**) time spent immobile; (**D**) freezing time (WT, *n* = 11; *Col6a1*$^{-/-}$ *n* = 9). Error bars indicate s.e.m.; insets report cumulative data (a, c, d) or average (b). WT, wild-type.

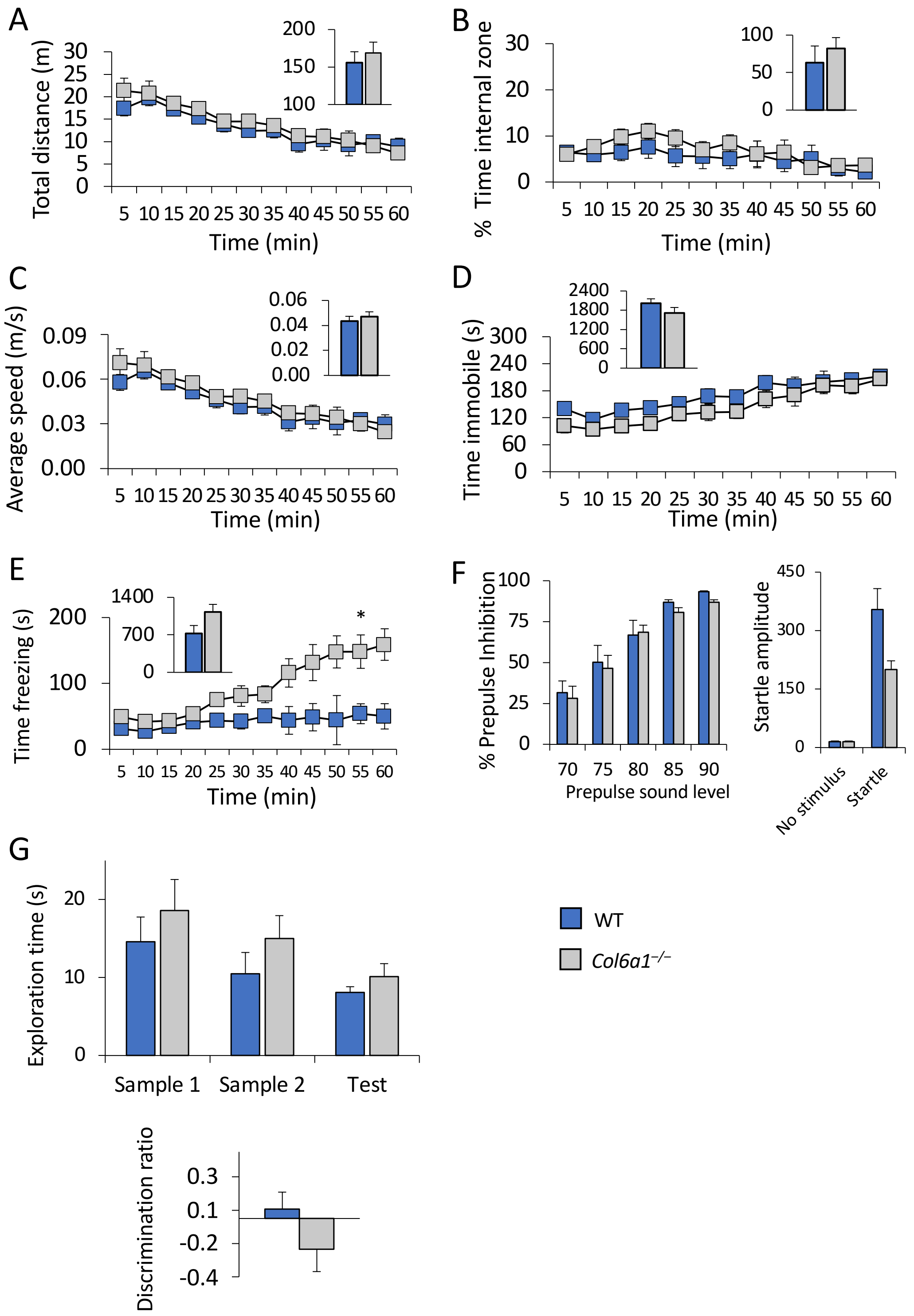

Supplementary Figure S2

Fig. S2. Behavioral tests in 12-month-old mice. Results of different behavioral analyses conducted in 12-month-old wild-type and *Col6a1*$^{-/-}$ mice. (**A-E**) Open field arena. (**F**) Prepulse inhibition. (**G**) Novel object recognition. (*, $P < 0{,}05$; WT, $n = 11$; *Col6a1*$^{-/-}$ $n = 9$). Error bars indicate s.e.m. WT, wild-type.

Disease Models & Mechanisms • Supplementary information

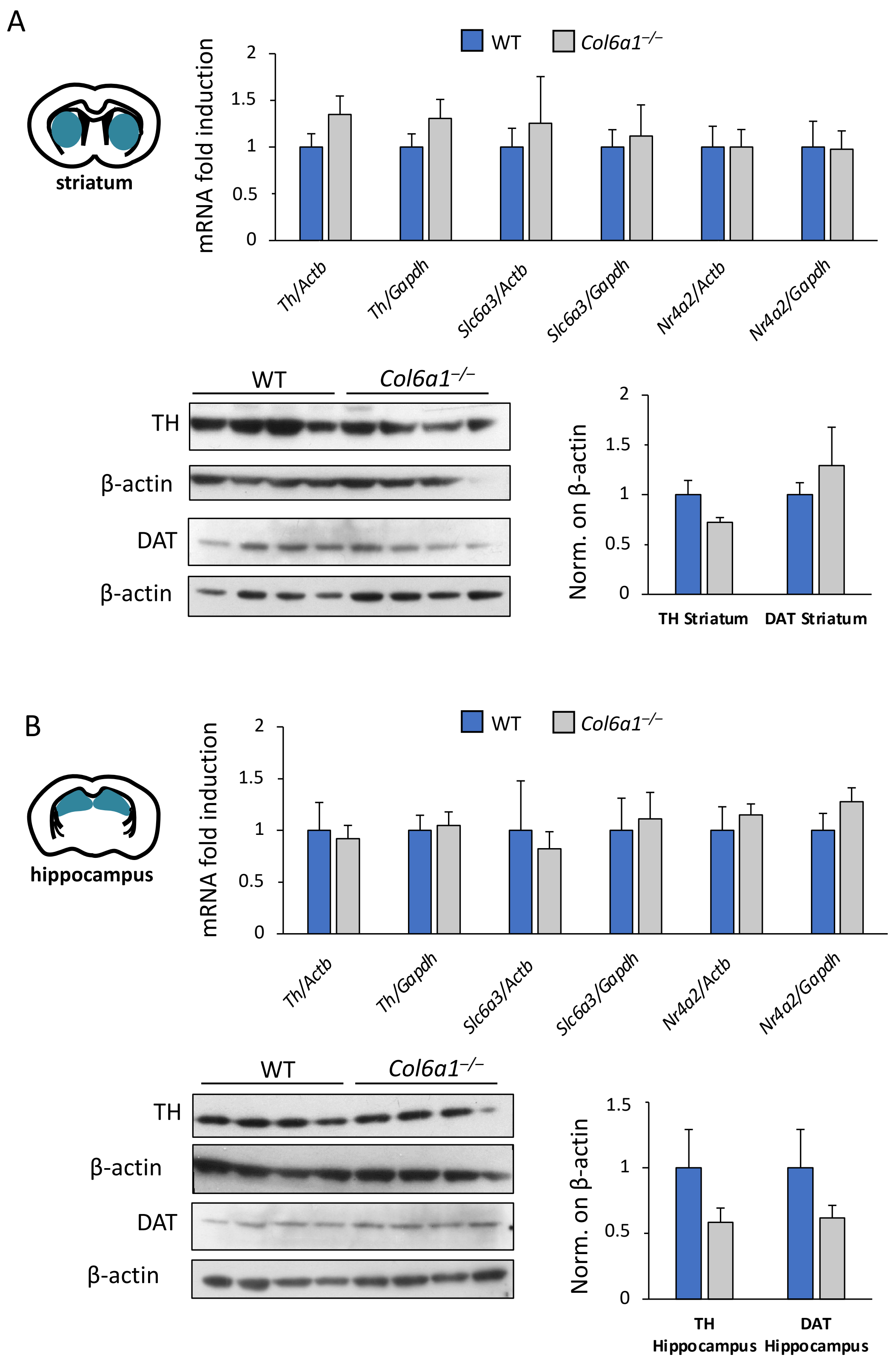

Supplementary Figure S3

Fig. S3. Dopaminergic signaling in *Col6a1*$^{-/-}$ striatum and hippocampus. **(A)** qRT-PCR of analysis for *Th*, *Slc6a3* and *Nr4a2* mRNA levels (upper panel) and of TH and DAT protein levels in the striatum of wild-type and *Col6a1*$^{-/-}$ mice. (**B**) qRT-PCR of analysis for *Th*, *Slc6a3* and *Nr4a2* mRNA levels (upper panel) and of TH and DAT protein levels (lower panel) in the hippocampus of wild-type and *Col6a1*$^{-/-}$ mice. (*, $P < 0.05$; unpaired two-tailed Student's t test; $n = 12$ mice each genotype). Error bars indicate s.e.m. WT, wild-type.

Disease Models & Mechanisms • Supplementary information

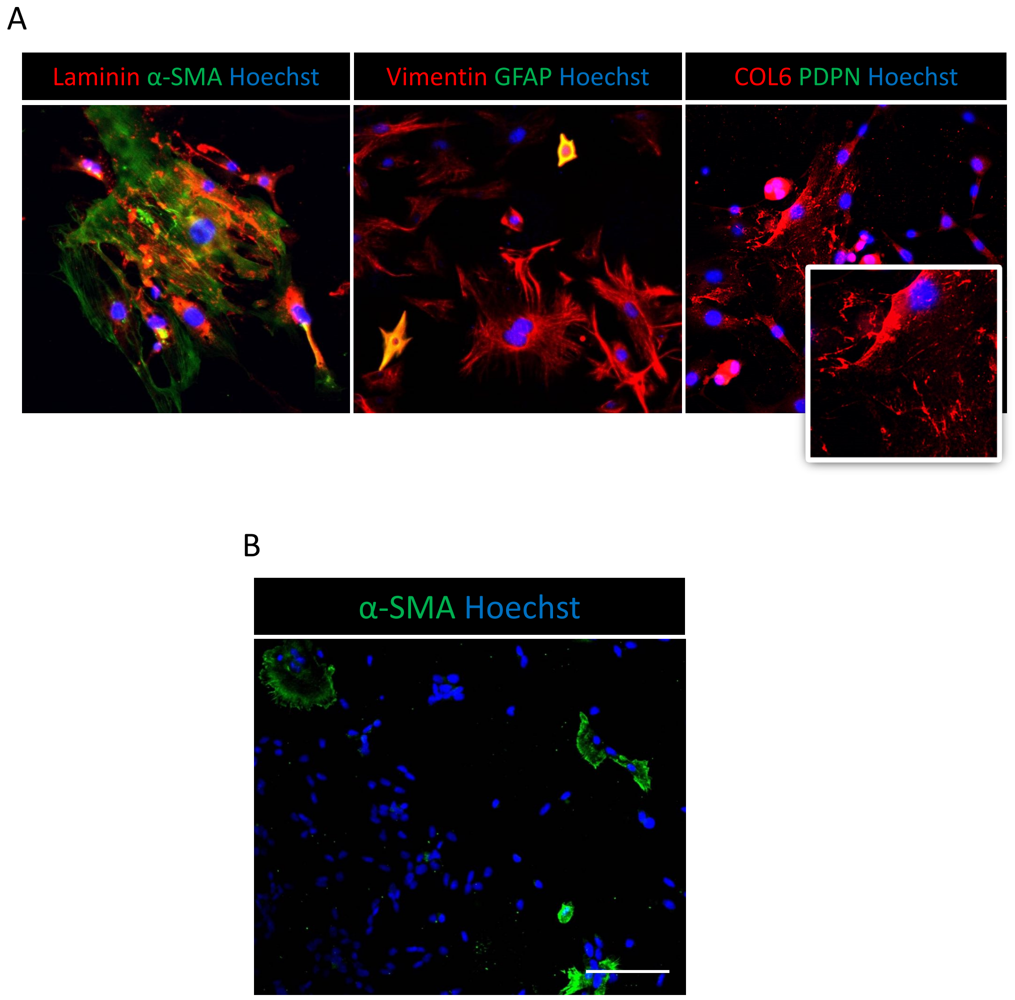

Supplementary Figure S4

Fig. S4. Meningeal cell cultures from wild-type mice produce COL6. (**A)** Immunohistochemistry analysis for various markers in primary meningeal cell cultures derived from wild-type mouse pups. The cultures express the fibroblast markers vimentin and α-SMA, but do not express the astrocyte marker GFAP and the endothelial marker podoplanin (PDPN). COL6 deposition is also observed (right panel and inset). (**B)** Immunohistochemistry for vimentin, performed on co-cultured meningeal and SH-SY5Y cells. Meningeal cells are positive for α-SMA (green), while SH-SY5Y cells are negative for the marker. Scale bar, 100 μm.

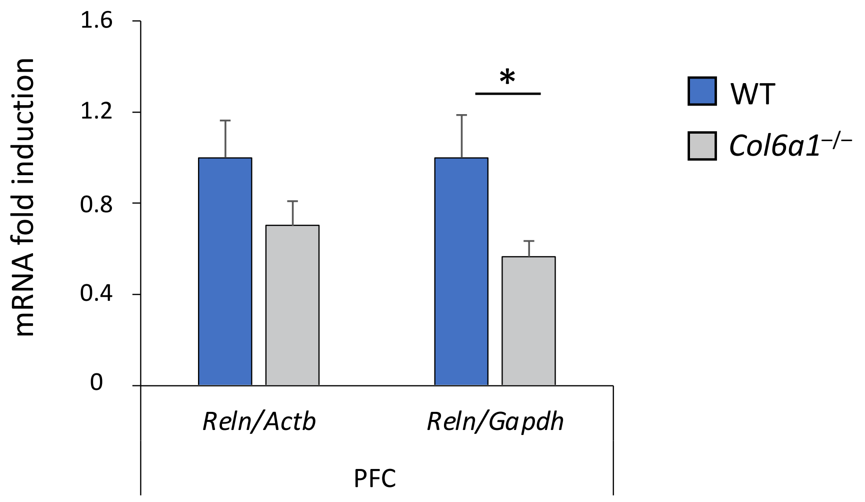

Supplementary Figure S5

Fig. S5. *Reln* expression in wild-type and *Col6a1*$^{-/-}$ prefrontal cortex. qRT-PCR of analysis for *Reln* mRNA levels in the medial prefrontal cortex (PFC) of wild-type and *Col6a1*$^{-/-}$ mice. (*, $P < 0.05$; unpaired two-tailed Student's t test; $n = 12$ mice each genotype). Error bars indicate s.e.m. WT, wild-type.

Disease Models & Mechanisms • Supplementary information

Table S1. List of primers used for qRT-PCR.

GENE	SPECIES	FORWARD PRIMER	REVERSE PRIMER
Actb	Mouse	CTAAGGCCAACCGTGAAAAG	ACCAGAGGCATACAGGGACA
Drd2	Mouse	ATCTCTTGCCCACTGCTCTTTGGA	ATAGACCAGCAGGGTGACGATGAA
Gabbr1	Mouse	ATTTCCGATGTGACCCCGAC	TTCGATTCACCTGGCAGTGG
Gapdh	Mouse	ATCCTGCACCACCAACTGCT	GGGCCATCCACAGTCTTGTG
GAPDH	Human	CACCATCTTCCAGGAGCGAG	CCTTCTCCATGGTGGTGAAGAC
Grin2a	Mouse	TCCTTGAACCCTTCAGCGCC	AGAAGGCCCATGGGGAGCTTT
Grin2b	Mouse	CTGAACCTCCTGTGTGAGAGGAAA	ATTCGGATGCCAGCTCCGCT
Htr1a	Mouse	CTGTTTATCGCCCTGGATG	ATGAGCCAAGTGAGCGAGAT
Nurr1	Mouse	CAACTACAGCACAGGCTACGA	GCATCTGAATGTCTTCTACCTTAATG
Pitx3	Mouse	GCAACTGGCCGCCCAAGG	AGGCCCCACGTTGACCGA
Slc6a3	Mouse	ATCAACCCACCGCAGACACCAGT	GGCATCCCGGCAATAACCAT
SLC6A3	Human	GCCTGCTTGCTGATATTGCAGT	TGGCCAACATCCTTCACTCA
Th	Mouse	CCTTTGACCCAGACACACAGCA	ATACGAGAGGCATAGTTCCTGAG
TH	Human	GCCCTACCAAGACCAGACGTA	CGTGAGGCATAGCTCCTGA

Table S2. Clinical features of BM/UCMD patients.

Patient #	Phenotype	Gene	Nucleotide	Amino Acid	Age at onset	Symptoms at onset	Best motor function	Age at LoA (yrs)	Age at NIMV (yrs)	Age at last visit	Contractures	Hyperlaxity	Scoliosis
1, M	UCMD	*COL6A3*	c.6210+1G>A	p.Asp2022_Lys2052del	Birth	Hip dysplasia, multiple contractures	Walking with support	5	10	20	Yes	Yes	Yes
4, F	UCMD intermediate	*COL6A2*	c.954G>A*	p.Lys318Lys	Birth	Hip dysplasia, multiple contractures	Walking	10	14	22	Yes	Yes	Yes
5, M	UCMD intermediate	*COL6A1*	c.841G>A	p.Gly281Arg	Birth	Hip dysplasia, multiple contractures, cataract	Walking	14	17	29	Yes	No	Yes
8, M	BM de novo	*COL6A3*	c.4859C>T	p.Pro1620Leu	Birth	Hypotonia, finger contractures	Run	No	No	18	Yes	No	No
9, F*	BM AD	*COL6A3*	c.5035G>T	p.Gly1679Trp	Adolescence	Lower limb weakness, difficulty climbing stairs	Run	No	No	54	Yes	No	No
11, F*	BM AD	*COL6A3*	c.5035G>T	p.Gly1679Trp	Adolescence	Shoulder and knee dislocation	Run	No	No	47	Yes	No	No
12, F*	BM AD	*COL6A3*	c.5035G>T	p.Gly1679Trp	Adulthood	Muscle weakness and pain in the upper limb	Run	No	No	42	No	No	No
14, F*	BM AD	*COL6A3*	c.5035G>T	p.Gly1679Trp	Adolescence	Muscle weakness in the upper limb	Run	No	No	20	No	No	No

Related patients are indicated with an asterisk. AD, autosomal dominant; F, female; LoA, loss of ambulation; M, male; NIMV, non-invasive mechanical ventilation; yrs, years.

Disease Models & Mechanisms • Supplementary information
